# Self-healing capacity of nuclear glass observed by NMR spectroscopy

**DOI:** 10.1038/srep25499

**Published:** 2016-05-05

**Authors:** Thibault Charpentier, Laura Martel, Anamul H. Mir, Joseph Somers, Christophe Jégou, Sylvain Peuget

**Affiliations:** 1 NIMBE, CEA, CNRS, Université Paris-Saclay, CEA Saclay 91191 Gif-sur-Yvette, France; 2 European Commission, Joint Research Centre (JRC), Institute for Transuranium Elements (ITU), Postfach 2340,D-76125 Karlsruhe, Germany; 3 CEA, DEN, DTCD, SECM, Laboratoire d’Étude des Matériaux et Procédés Actif, 30207 Bagnols-sur-Cèze, France

## Abstract

Safe management of high level nuclear waste is a worldwide significant issue for which vitrification has been selected by many countries. There exists a crucial need for improving our understanding of the ageing of the glass under irradiation. While external irradiation by ions provides a rapid simulation of damage induced by alpha decays, short lived actinide doping is more representative of the reality. Here, we report radiological NMR experiments to compare the damage in International Simplified Glass (ISG) when irradiated by these two methods. In the 0.1 mole percent ^244^Cm doped glass, accumulation of high alpha decay only shows small modifications of the local structure, in sharp contrast to heavy ion irradiation. These results reveal the ability of the alpha particle to partially repair the damage generated by the heavy recoil nuclei highlighting the radiation resistance of nuclear glass and the difficulty to accurately simulate its behaviour by single ion beam irradiations.

Borosilicate glasses, besides other numerous applications, have been recognized as valuable materials for the conditioning of nuclear wastes^1^. Among the long term behavior issues, the structural evolution of the glass under irradiation (beta and alpha decays)^2^ is of primary importance, along with its chemical durability in a deep-geological repository^3^,^4^. A major difficulty in its safety assessment is the development of accurate methods to predict its long term behavior based solely on short term investigations. The two main experimental approaches employed are : i) external irradiation with ions at a fluence level simulating the aging of the nuclear waste glass over its life time; ii) doping with short-lived radioactive isotopes (^244^Cm, ^238^Pu, ^234^Cs …) to generate a lifetime decay-dose in a span of few years^2^. The first approach is more flexible as different kinds of irradiations can be used to investigate separately the effects of both electronic and nuclear stopping powers. Nevertheless, the full representativeness of the true ageing conditions is debatable^2^. Heavy ion irradiation, simulating damage induced by the recoil nuclei (RN) of alpha decays (αD) indicates important structural transformations of the glass network with a change in boron and aluminum coordination numbers and an increase in the glass depolymerization^5^,^6^,^7^. These observations have been correlated to other structural transformations observed by Raman spectroscopy and Molecular Dynamic simulation (MD) studies^7^,^8^ and in the evolution of various macroscopic properties^7^. A model of irradiation induced vitrification involving ballistic disordering and fast quenching events has been proposed to explain the glass restructuration under αD irradiation^9^. Therein, the glass structure is disordered by the displacements cascades arising from RN and is then relaxed to a new glassy state in the ensuing very rapid thermal quench. It suggests that the nuclear damage generated by RN controls the glass restructuration under αD^7^. Nevertheless, recent results using sequential and simultaneous ion irradiations^10^,^11^ have shown that the electronic energy loss of the alpha particle (AP) induces partial repair of the damage generated by heavy ions in simple and complex borosilicate glasses. The ionization induced recovery has also been observed during sequential irradiation of SiC^12^ wherein the degree of defect recovery was observed to increase with increase in the electronic stopping power of the ion. In glasses as well as in SiC, ionization induced thermal spike and defect recovery as a result has been proposed to be the responsible mechanism^12^,^13^. These results suggest that such a defect recovery process should also be effective in actinide bearing glasses due to irradiation of the glass by AP and RN. Such coupled effects between nuclear and electronic stopping power have also been observed for other nuclear materials when subjected to external irradiation^14^,^15^,^16^,^17^,^18^,^19^,^20^ but to date no direct experimental proof of this coupling occurring in the actual nuclear waste glass has been provided.

The actinide doping approach, despite being more realistic, limits the number of structural characterization techniques that can be deployed, simply due to the radioactivity level of the sample. Among spectroscopic techniques, solid state NMR is certainly the one that has seen many major achievements in the last few decades and is nowadays considered as a cutting-edge method for the elucidation of glass structure^21^,^22^. ^29^Si MAS NMR was used to characterize damage in Pu-bearing zircon^23^, and a broad signal arising from the amorphized volume could be detected at levels far beyond that achievable with usual macroscopic measurements^24^. In this work, MAS NMR housed in a glove-box (see Martel *et al*.^25^) enabled very high sample spinning-frequency (40–50 kHz) using standard MAS NMR containers (1.3 mm outer diameter). ^29^Si, ^23^Na, ^11^B and ^27^Al could then be probed in old ^244^Cm doped glasses, enabling the investigation of the nature of the coupling effect between AP and RN in nuclear glass by comparison of these results with those obtained using heavy ion irradiation of glasses (Xe and Au ions with respective energies of 92 MeV and 1 to 7 MeV, see^6^,^7^). The International Simplified Glass (ISG) has been chosen for this study as it is considered by the international community as a good surrogate of nuclear borosilicate glasses developed worldwide^3^. Two old ^244^Cm doped ISG glass (ISG-Cm) samples were analyzed by NMR; one stored since its fabrication in 2007 had accumulated an αD dose of 4.4 × 10^18^α/g (equivalent to 10000 years of disposal); the second was taken from the former but was annealed at 873K for 2h and the temperature was then decreased up to room temperature with a slope of 1K/min to fully recover the irradiation induced damage. Calorimetric study of ^244^Cm doped glass^9^ has in fact showed that an annealing of the damaged glass above transition temperature for a few minutes is sufficient to induce a complete recovery of the damage. This annealing protocol was chosen to reproduce the thermal cycle during which the glass rod was fabricated in 2007, so as to have the same cooling rate in the transition range and then to stabilize the same structure of the glass as during the fabrication process (the thermal history of the glass is known to change its structural state^5^). These two samples were then characterized periodically for 22 months (see Table S1 in the Supplementary Material), during which their αD dose evolved from 4.4 to 5.8 × 10^18^α/g and 0 to 1.4 × 10^18^α/g, respectively.

The ^11^B MAS NMR spectra, presented in Fig. 1 (upper panel for Cm doped and lower panel for ion irradiated glass, the latter have been acquired at a higher magnetic field – see experimental section), are characterized by two main peaks attributed to the four coordinate boron, BO_4_, at 0 ppm and three coordinated boron, BO_3_, at 10 ppm^26^. The two dimensional multiple quantum MAS (MQMAS) spectra (see Supplementary Information, Fig. S1) show that standard deconvolution into BO_4_ and BO_3_ units can be applied to the spectra of ISG-Cm glasses to extract the BO_3_ and BO_4_ populations, using two components for each speciation (see Supplementary Information for details). We intentionally do not provide any further discussion here on this partitioning as it is subjected to higher uncertainty at the moderate magnetic field used here (9.4T), even if supported by higher field (11.75T) two-dimensional experiments^6^ which provided the initial NMR parameters used in the fitting procedure. The red spectrum in the upper panel of Fig. 1 corresponds to the Cm doped glass (ISG-Cm-annealed) which was annealed and then characterized after a time period of one month during which 7 × 10^16^ alpha/gram was accumulated. It was shown by several studies summarized in^7^ that such a low alpha decay dose is not enough to induce any measurable density and fictive temperature variation and therefore does not induce any significant measurable structural changes. Therefore, it will be regarded as the undamaged reference spectrum for the Cm doped glass. A comparison of this spectrum with the aged glass spectrum (ISG-Cm-damaged shown in blue) shows an alpha decay induced increase of BO_3_ by about 7%. No significant variation in longitudinal relaxation times or line widths is observed (see Supplementary Information).

^23^Na MAS and MQMAS spectra show a single broad line which narrows slightly after annealing (see Supplementary Information). For ^29^Si, there is a main peak at −95 ppm indicative of relatively high polymerized glass^6^. Despite its low sensitivity (natural abundance of 4.68%) leading to noisy ^29^Si spectra, no significant difference for aged and annealed glasses could be found (see Supplementary Information).

The ^27^Al MAS NMR spectrum of the annealed glass at an αD dose of 7 × 10^16^α/g is defined by one main peak corresponding to four-fold coordinated Al (AlO_4_). MQMAS spectroscopy did not revealed the formation of highly coordinated species in ISG-Cm glass (damaged and annealed), as recently found in external heavy ion irradiation studies^6^ (Supplementary Information Fig. S10). A significant difference is observed between the ^27^Al MAS NMR spectrum of the annealed glass and those of the damaged glass (αD doses of 4.4 and 5.8 × 10^18^α/g) (Fig. 2, upper panel). There is a continuous variation with alpha decay dose of the ^27^Al spectrum over the period of 22 months after annealing the initially damaged glass. The final spectrum after 22 months (αD dose of 1,4 × 10^18^α/g) is very similar to those of the ISG-Cm-damaged glass with an initial αD dose of 4.4  × 10^18^α/g and which increased to 5.8 × 10^18^α/g over a 10 months time period. No significant spectral evolution was observed in going from 4.4  × 10^18^α/g to 5.8  × 10^18^α/g indicating a saturated damaged state at 1.4 × 10^18^α/g already. Because MQMAS spectroscopy do not show any evidence of highly coordinated species, the width increase from the pristine state arises mainly from an increase of the local electric field gradient (EFG) and can have several origins, i.e. an increase of the NMR parameter distribution width either related to a change in the charge compensator of the AlO_4_ tetrahedra (here Ca^++^ or Na^+^)^27^ or/and an increase of the local disorder around AlO_4_ tetrahedra. In such oxide glass compositions, the Na ions are considered as the main charge compensators of aluminum tetrahedral (peak position at −20 ppm). It is also known that the charge compensation by Ca ions (i.e. Na substitution) induces a very important increase of the width of Al spectra^27^. Therefore, the augmentation of Ca ion population participating into the charge compensation mechanism of AlO_4_ units is one of the probable mechanism to explain the increase of the width of ^27^Al MAS NMR spectrum, as the ballistic disordering is known to induce significant atomic displacements and that Na ions are the most displaced atoms in the glassy structure^28^. Therefore it is possible that a part of the Na atoms are ejected from the vicinity of AlO_4_ tetrahedra and replaced by Ca ions. If the increase of the global disorder around AlO_4_ tetrahedra due to the global damage of the glassy network could certainly contribute to the width increase, as no such broadening was observed for the other former cations in the glass; here boron and silicon; we believe that change of charge compensator is the main mechanism for explaining this width increase.

Figure 3 shows the variation of the width of the aluminum spectra and of BO_4_ population versus αD dose. A regular evolution of the structure with dose with a saturation phenomenon occurring at around 2 × 10^18^α/g is found, and can be fitted with a direct impact model^7^, f_a_ = 1-exp(-v_i_D_α_), where f_a_ is the damage volume fraction, v_i_ is the damage volume per event (g) and D_α_ is the alpha decay dose per gram. Such an evolution indicates that only a single alpha decay event is enough to induce a full transformation of the material to the saturated damaged state. This model is usually used in glassy materials to fit the evolution of the density^2^,^7^, mechanical properties^7^ and fictive temperature^9^ that are macroscopic parameters. Nevertheless, this model can also be used to adjust the variation of structural parameters obtained from NMR spectroscopy considering that NMR probes a macroscopic volume and offers then a mean evaluation of the local structural changes around atoms that are proportional to the damage volume fraction. From the fit performed, a damage volume of around 480 nm^3^ associated to an individual αD event was extracted which is in quite good agreement with those obtained (~300 nm^3^) by fitting evolution of macroscopic or structural parameters of other actinides doped glasses^7^,^9^,^29^. Likewise, MD simulations of displacement cascades, representative of the RN damage, indicate a damage volume of about 270 nm^3^,^29^. The similar damaged volume with MD result, that model only the RN damage, suggest that these spectral variations with the dose are controlled by the accumulation of ballistic damage induced by the RN.

These structural modifications (decrease of the average boron coordination number and increase of the ^27^Al local EFG) are qualitatively similar to those observed in glasses submitted to external heavy ion irradiation (1 to 7 MeV Au ions or 92 MeV Xe ions)^6^,^7^. Nevertheless, self-irradiation by αD induces much smaller change of the boron coordination, i.e. +7% by ^244^Cm doping compared to +16% by Xe or +13% by Au ions. Similarly, ^27^Al MAS NMR data from ISG-Cm only exhibit four coordinated Al atoms whereas around 4 and 2% (8 and 4%) of penta and hexa coordinated aluminum atoms were measured in Xe (Au, respectively) irradiated ISG glass^6^,^7^. Moreover, no significant depolymerization of ISG-Cm is observed in ^29^Si and ^23^Na spectra contrary to the results obtained on Xe and Au ISG irradiated glass^6^,^7^ (see Supplementary Information Figs S5 and S9). Several origins of this lower damage state in ISG-Cm could be considered, a chemical effect due to the transmutation of ^244^Cm into ^240^Pu, a difference between the damage efficiency of Au (1 to 7 MeV)/Xe 92MeV ions and ^244^Cm alpha decay RN or an annealing effect of AP on the damaged state generated by RN. During the eight years of storage of ISG-Cm glass, around 25% of the Cm atoms have been transmuted to Pu atoms. So the chemical effect associated to the transmutation of Cm into Pu concerns only 0.025at%, whose role in the glass structure is quite similar (modifier role with coordination number between 7 and 8)^30^ and therefore cannot significantly affect the glassy structure to explain such differences in boron coordination number. Another possibility to explain this lower damage level in ISG-Cm could be related to a difference between the damage state generated by Au/Xe ions and the one generated by heavy recoil nuclei of ^244^Cm alpha decays. But it was shown by Raman spectroscopy, micro hardness and swelling that swift heavy ions with electronic energy loss at least greater than 4 keV/nm (case of Xe irradiation) cause a significant damage level which is in good qualitative and quantitative agreement with the damage from the ballistic collisions, the case of heavy RN of ^244^Cm alpha decays^11^. Therefore this lower damage state is most likely a result of the simultaneous irradiation with RN and AP in ^244^Cm alpha decays, which mainly undergo nuclear and electronic collisions respectively. Indeed, competition between these two energy loss mechanisms and its impact on the global damage level has been addressed recently in a sodium borosilicate glass using double ion beam irradiations^10^. Mono and double beam irradiation experiments on simple borosilicate glasses with ions representative of alpha particles (2 MeV He) and recoil nuclei (14 MeV gold ions) showed that AP causes a partial damage repair of the pre-existing defects. The studies^10^,^11^ have shown that electronic stopping power at least up to 1.5 keV/nm can cause a recovery of the pre-existing damage induced either by swift heavy ions like 92 MeV Xe ions or low (~1 MeV) to intermediate energy (~14 MeV) heavy ions like gold ions. Therefore, the lower damage state of the curium doped ISG compared to that of the heavy ions irradiated ISG glasses also suggest that such a competition exists during αD irradiation, with a partial repair of the recoil nuclei damage by AP. This competitive mechanism can be understood in the framework of the unified thermal spike phenomenology^31^. Under this phenomenology, the energy loss of the ions causes local heating around the ion path and in certain cases can induce local melting and ion track formation (the local temperature depends on the energy loss of the ions). This mechanism has been used to describe the ion track formation during 92MeV Xe ion irradiation (high electronic stopping power)^6^ and 1 to 7 MeV Au ion irradiation (high nuclear stopping power)^31^. This thermal spike description is in full agreement with the modifications of the local order observed in borosilicate glass under such irradiation conditions^6^,^7^ mainly a decrease of boron coordination number and an increase of non-bridging oxygen concentration. Indeed in borosilicate glasses, it is well known that the increase of the temperature induces a shift of the reaction BO_4 _↔ BO_3 _+ NBO to the right^32^ and so favors trigonal boron and NBO. In the case of ISG-Cm, however, RN and AP simultaneously irradiate the material. The RN (just as for Au) induce very high pseudo-temperature inside the displacement cascades^29^ (which can be qualified as ballistic melting in the framework of unified thermal spike phenomenology), higher than the glass melting temperature, which results in a similar glass transformation just as for Au or Xe irradiation. On the contrary, 99% of AP energy is lost in electronic collisions with a very low electronic stopping power of around 0.5 keV/nm, which is insufficient, according to the thermal spike calculation^10^,^13^, to reach the glass melting temperature and then generate the same damage level as heavy ions. Nevertheless, temperatures of around 600 K can be reached^10^ which are high enough to induce a partial repair of the irradiated glass structure, as has been shown by numerous calorimetric experiments performed on actinides doped glasses 2,9,33. So the partial repair of the glassy structure can occur via defect recombination activated by the AP thermal spike, which does not need to exceed the glass transition temperature but rather activate the defect recombination. Therefore the diminished damage level observed in the ISG-Cm can be explained by the competitive effect between damage generation by the RN and partial damage repair due to AP.

## Conclusion

The first MAS NMR experiments on radioactive glasses, close to the reality and doped with ^244^Cm have revealed similar transformations as identified when samples are subjected to external irradiation by heavy ions, but to a much lower extent. This clearly demonstrates a competitive effect occurring between the recoil nucleus and the alpha particle emitted during alpha decay. The partial damage repair occurs due to alpha particle irradiation of the recoil nucleus pre-damaged regions. Deeper investigations of such coupled effects will be of great importance in the near future to assess the appropriateness of accelerated experiments to simulate the ageing of nuclear materials under working conditions.

## Methods

### Sample preparation

In 2007, one batch of ^244^Cm doped ISG glass (ISG-Cm) was fabricated in the Atalante laboratory whose composition (molar %) is SiO_2_ 60.02, B_2_O_3_ 15.96, Na_2_O 12.61, Al_2_O_3_ 3.81, CaO 5.69, ZrO_2_ 1.71, Cm_2_O_3_ 0.13, PuO_2_ 0.07. This glass is considered as a good surrogate to the French R7T7 industrial glass and was chosen by the international community as an international glass standard to compare the long term behavior methodologies developed worldwide^34^. The glass was produced by a melting process at 1673K and quenched to a temperature slightly above the glass transition temperature (873K) and cooled more slowly (1K/min) to remove any residual stresses. Similar protocol as described in^29^ was applied to evaluate the glass homogeneity and chemical composition by using optical microscopy, SEM, XRD, gamma scanning and calorimetric experiments (to determine accurately the curium content of the glass). In March 2013, two glass powders were prepared, one from the damaged glass stored since 2007 with an accumulated αD dose of 4.4 × 10^18^α/g and one from the same glass, but annealed at 873K for 2 h and then cooled down slowly (1K/min) to fully recover the irradiation induced damage. These two powders were then characterized periodically during which their αD dose evolved from 4.4 to 5.8 × 10^18^α/g (10 months) and 0 to 1.4 × 10^18^α/g (22 months), respectively.

### NMR measurements

All spectra of the ISG-Cm glasses were collected on a 400WB Bruker Avance III spectrometer operating at a magnetic field of 9.4T with spinning frequencies from 40 to 50 kHz using 1.3mm outer diameter rotors (30mg of powder for each glass sample was packed in rotors). In an earlier approach developed by Farnan *et al*.^23^ a triple containment barrier for MAS rotors of large outer diameter (7mm) were used, at the expense of the spinning frequency (slow rotation) and the filling factor. Here, an alternative approach was deployed, as adopted by Martel *et al*.^25^. At JRC-ITU a glove-box was designed to confine the whole MAS NMR probe inside the magnet bore so that the former could be employed at its nominal performance. As an example, frequencies up to 65 kHz could be reached^35^. ^27^Al, ^11^B and ^23^Na spectra were acquired with single pulse excitation of short lengths (1usec, tip angle ~10–20°) to ensure quantitatively and with recycle delays ensuring a full relaxation of the nuclear spins (0.5s for ^23^Na and ^27^Al, 2s for ^11^B). Multiple Quantum MAS (MQMAS) spectra were collected using the Z-filter pulse sequence (for ^27^Al) or the RIACT (II) pulse sequence for ^23^Na and ^11^B^26^. For ^29^Si, the CPMG pulse sequence has been employed, coadding echoes, followed by Gaussian apodization of 100 Hz before Fourier Transform Data were processed and fitted with an in-house written code^26^. Other experimental data are gathered in the Supplementary Information.

For purpose of comparison, spectra collected on the pristine and heavy Ion (Xe and Au) irradiated ISG glasses are also presented. They were acquired on a conventional 500WB Bruker Avance II spectrometer operating at a magnetic field of 11.75~T as described in ref. 6. Because of the limited access to the radioactive NMR facility (and loss of samples once incorporated in the glove box), it was not possible to repeat MAS NMR experiments for these under the same experimental conditions.

## Additional Information

**How to cite this article**: Charpentier, T. *et al*. Self-healing capacity of nuclear glass observed by NMR spectroscopy. *Sci. Rep*. **6**, 25499; doi: 10.1038/srep25499 (2016).

## Supplementary Material

Supplementary Information

## Figures and Tables

**Figure 1:**
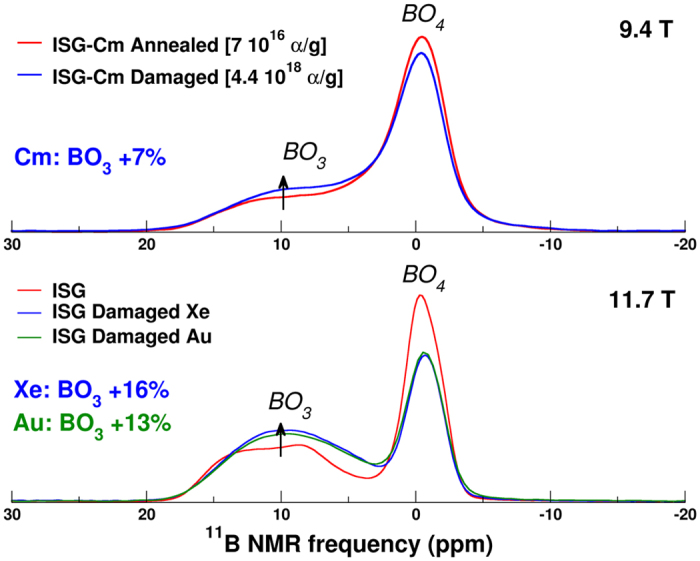
Upper Panel: Comparison between the ^11^B MAS NMR spectra (9.4T) of the damaged and annealed ISG-Cm glasses. Lower Panel: Comparison between the ISG and ISG-damaged with Xe (92MeV) and Au (1–7MeV) irradiation^6^,^7^ acquired at higher magnetic field (11.7T). Spectra are normalized to the same area.

**Figure 2:**
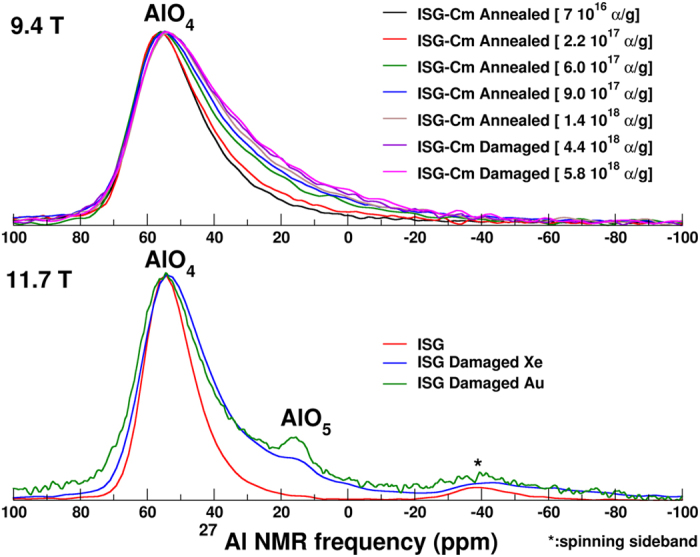
Upper Panel: ^27^Al MAS NMR spectra of the ISG-Cm glasses (9.4T). Lower Panel: ^27^Al MAS NMR spectra of the ISG and ISG-damaged Xe (92MeV) and Au (1–7MeV) irradiation from^6^,^7^ (11.7T). Spectra have been normalized to the same height.

**Figure 3:**
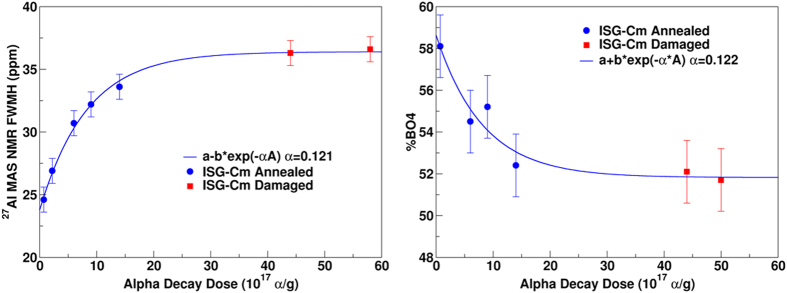
Left: Variation of the full width at half maximum (FWHM) of the ^27^Al MAS NMR spectra with the alpha decay dose. Right: Variation of the fraction of the BO_4_ units with the alpha decay dose. Solid lines show the fit of a single exponential to the data.
