# Supplementary material for: Self-healing capacity of nuclear glass observed by NMR spectroscopy
Source: Sci Rep. 2016 May 5;6:25499. doi: 10.1038/srep25499 (PMC4857743; doi:10.1038/srep25499)
Supplement: Supplementary Information [file srep25499-s1.pdf]

Self-healing capacity of nuclear glass observed by NMR spectroscopy

Thibault Charpentier[1,*], Laura Martel[2], Anamul H. Mir[3], Joseph Somers[2], Christophe Jégou[3] and Sylvain Peuget[3,*]

[1] NIMBE, CEA, CNRS, Université Paris-Saclay, CEA Saclay 91191 Gif-sur-Yvette, France

[2] European Commission, Joint Research Centre (JRC), Institute for Transuranium Elements (ITU), Postfach 2340, D-76125 Karlsruhe, Germany

[3] CEA, DEN, DTCD, SECM, Laboratoire d'Étude des Matériaux et Procédés Actif, 30207 Bagnols-sur-Cèze, France

* Corresponding authors: thibault.charpentier@cea.fr, sylvain.peuget@cea.fr

1. Time and dose level

Time (Month)	Date	Dose level (α/g)	Glass
1	04/2013	$7\ 10^{16}$	ISG Annealed
4	07/2013	$2.2\ 10^{17}$	ISG Annealed
10	01/2014	$6.0\ 10^{17}$	ISG Annealed
15	06/2014	$9.0\ 10^{17}$	ISG Annealed
22	01/2015	$1.4\ 10^{18}$	ISG Annealed
72	04/2013	$4.4\ 10^{18}$	ISG Damaged
81	01/2014	$5.8\ 10^{18}$	ISG Damaged

Table S1: Time and dose level of ISG annealed and damaged glass characterized by NMR spectroscopy

2. ^{11}B NMR

^{11}B MAS NMR spectra were acquired with single pulse excitation of short length (1μs, tip angle ~ 10-20°) to ensure quantitatively and with a recycle delay ensuring a full relaxation of the nuclear spins (2s). Typically between 2048 and 8192 scans were accumulated to obtained good signal to noise ratio. Multiple Quantum MAS (MQMAS) spectra were collected using the two-pulse RIACT (II) pulse sequence [1] with pulse widths of 4μs and 5μs, at a RF field of ~150 kHz. Typically 480 transients were accumulated per t1-value with 48 rotor-synchronized t1 increment (20μs) and a recycle delay of 0.5s. MQMAS experiments had to be limited in duration as sample spinning out of working hours was not possible.

In order to assess the deconvolution (shown below) of the ^{11}B MAS NMR spectra into BO_3 and BO_4 units, Triple Quantum MAS experiments were performed (Fig. S1). As standard lineshapes were observed [1-3], the deconvolution of the ^{11}B MAS NMR spectra with four lines (2 BO_3 (e.g. 17 and 12 ppm) and 2 BO_4 (BO_4 surrounded by 4Si (-0.8±1.3 ppm) and by 3Si and 1B (1.5±1.6 ppm)), widely used in literature, (see Ref. [1] and reference therein) was performed, as shown in Fig. S2, yielding NMR parameters gathered in Table S2. To follow the effect of the dose rate on the structure, the sum between the two types of BO_4 will be considered as being more relevant. As shown in Fig. S3, no significant change in the ^{11}B NMR transverse and longitudinal relaxation times were observed under static (i.e. non spinning sample) conditions, showing that, despite the possible formation of paramagnetic center, relaxation behavior of the nuclei is only slightly modified in this radioactive material. In addition, it was check that absolute area of the spectrum was constant as shown in Fig. S4 so that no loss of signal is observed (invisible signal caused by paramagnetic centers). Variations of the spectrum area shows fluctuations of less than 10% and without trend (decrease or increase) so that we consider that a negligible part of the signal, if any, could be lost.

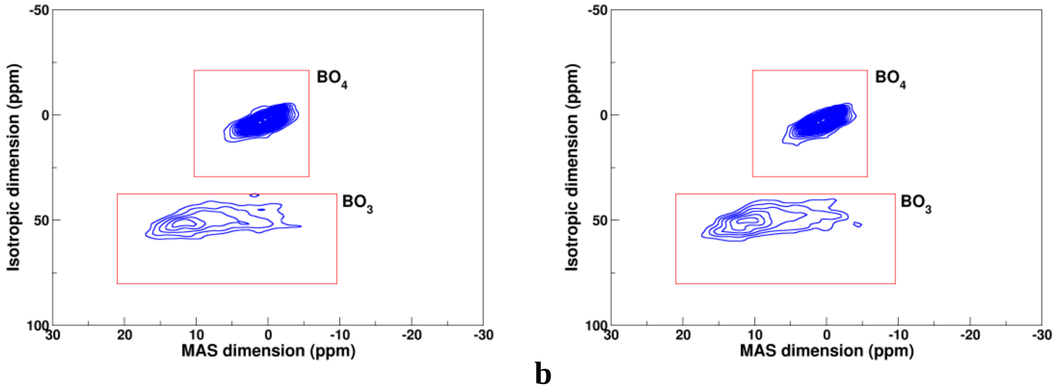

Figure S1: ^{11}B MQMAS spectrum of (a) the ISG-Cm annealed glass (7 10^{16} α/g) and (b) the ISG-Cm damaged glass (4.4 10^{18} α/g). The two types of boron species (e.g. BO_3 and BO_4) are clearly identified in the 2D map at standard positions in terms of NMR frequencies.

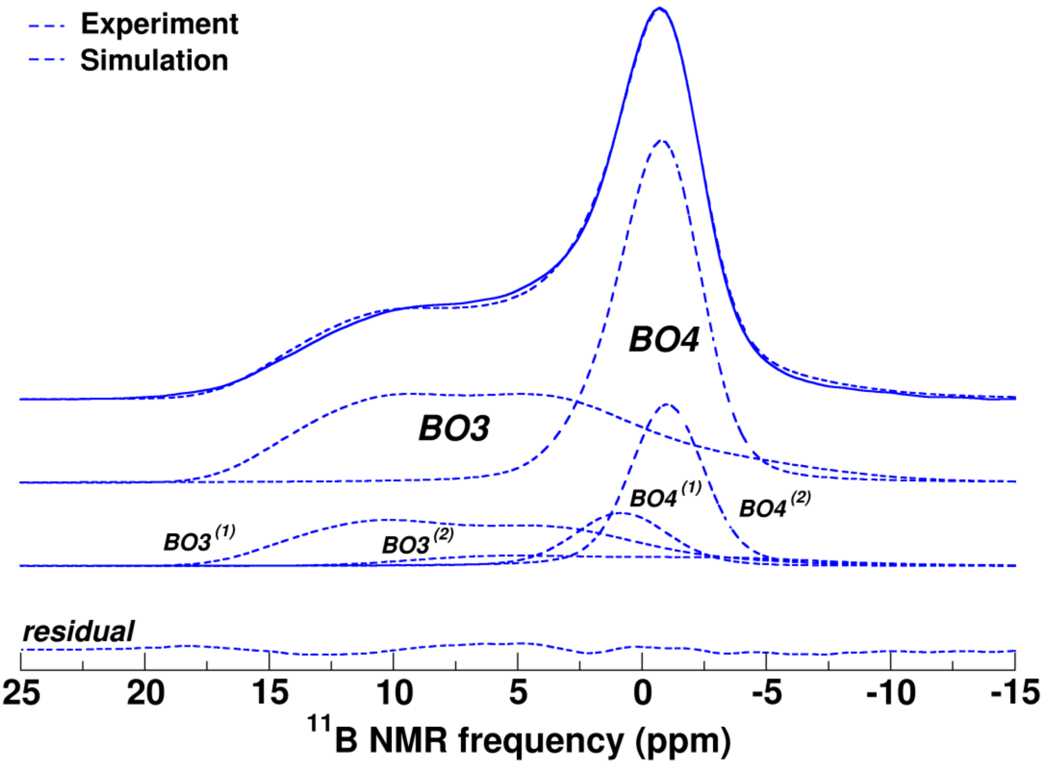

Figure S2: Example of ^{11}B MAS NMR spectrum analysis using the method described in [1]. NMR parameters are given in table S1. All ^{11}B MAS NMR spectra have been analyzed with the same NMR parameters (Table S1)

Site	δ_{iso} (ppm)	C_Q (MHz)	η
$BO_4^{(1)}$	1.5 (1.6)	0.6 (0.2)	0.6 (0.3)
$BO_4^{(2)}$	-0.8 (1.3)	0.2 (0.1)	0.6 (0.3)
$BO_3^{(1)}$	17 (1.7)	2.5 (0.1)	0.4 (0.1)
$BO_3^{(2)}$	12 (2.6)	2.7 (0.1)	0.2 (0.1)

Table S2: Mean values of ^{11}B NMR parameter distribution used to analyze the ^{11}B MAS NMR spectra. Standard deviation values are given in parentheses. δ_{iso}: isotropic chemical shift; C_Q : Quadrupolar coupling constant; η : quadrupolar asymmetry parameter.

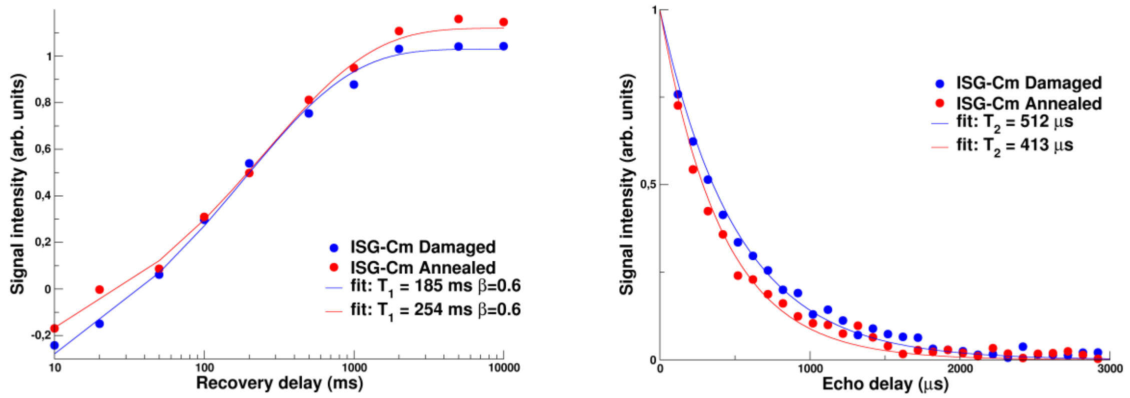

Figure S3: Longitudinal (left panel, using inversion-recovery pulse sequence) and transverse (right panel, using spin echo) ^{11}B relaxation times under static (i.e. non spinning) conditions with pulses selective on the central transition (-½ , ½). Inversion-Recovery and Spin-Echo curves were fitted with a stretched exponential $M(t) = M_\infty \times \left(1 - e^{-\left(\frac{t}{T_1}\right)^\beta}\right)$ and a single decaying exponential function $(t) = M(0) \times e^{-\frac{t}{T_2}}$, respectively.

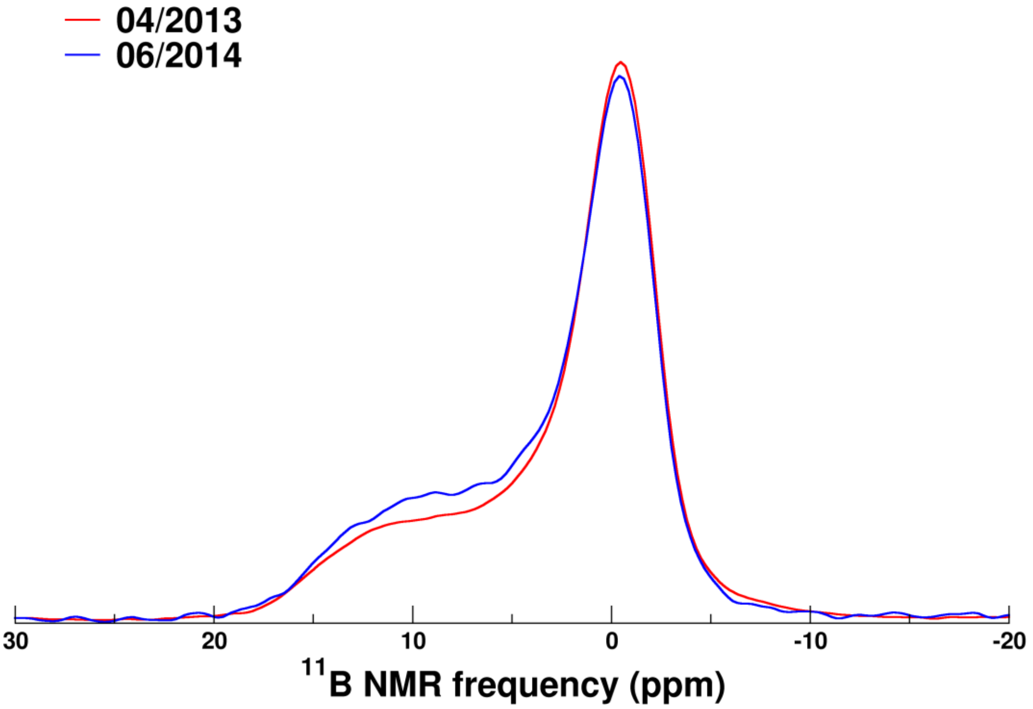

Figure S4: ^{11}B MAS NMR spectra of the ISG-Cm Glasses normalized to the same number of scans. The area are 2.4 10^6 and 2.6 10^6 (arbitrary units) for 04/2013 and 06/2014, respectively.

3. ^{23}Na MAS and MQMAS

^{23}Na MAS NMR spectra were acquired with single pulse excitation of short length (1μs, tip angle ~ 10-20°) to ensure quantitatively and with a recycle delay ensuring a full relaxation of the nuclear spins (0.5s). Typically between 2048 and 8192 scans were accumulated to obtained good signal to noise ratio. Multiple Quantum MAS (MQMAS) spectra were collected using the two-pulse RIACT (II) pulse sequence [1] with pulse widths of 4μs and 5μs, at a RF field of ~125 kHz. Typically 480 transients were accumulated per t1-value with 40 rotor-synchronized t1 increments (20μs) at a recycle delay of 0.5s.

^{23}Na MAS (Fig. S5) and MQMAS (Fig. S6) were performed on the two references glasses (ISG-Cm glasses at the beginning if this study) and with time evolution. As confirmed by the MQMAS experiments, the spectra of ISG-Cm Annealed (7 10^6 α/g) is defined by a single broad peak centered at about -20 ppm. Spectra were therefore analyzed using NMR parameter distribution (see [1] for details) yielding NMR parameters gathered in Table S3. With the dose there is a slight line broadening but less important than in the case of external ion beam irradiation [3]. For the ISG-Cm damaged glass, a small narrow peak was observed (dashed line in Fig. S5). The latter could be attributed to hydrated species at the surface [4], possibly here enhanced by air radiolysis occurring during the 7 years of storage. As shown in Fig. S7, no

significant change in the ^{23}Na NMR transverse and longitudinal relaxation times were observed under static (i.e. non spinning sample) conditions. With time, no further variations of the ^{23}Na MAS NMR spectrum was observed, as shown in Fig. S8.

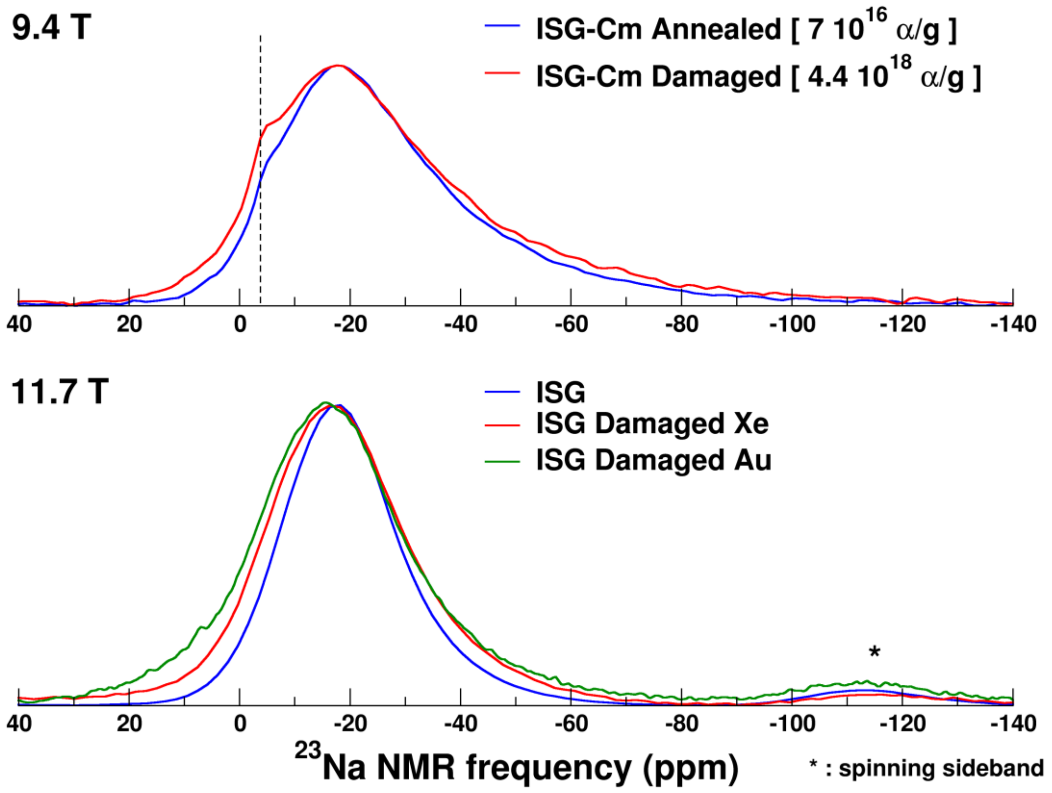

Figure S5: ^{23}Na MAS NMR spectra of (Upper panel) ISG-Cm glasses and (Lower panel) ISG and ISG damaged glasses by Xenon[3] and Gold [4] Irradiation. Dashed line indicates Na peak of hydrated surface species [5]. Spectra are normalized to the same maximum height.

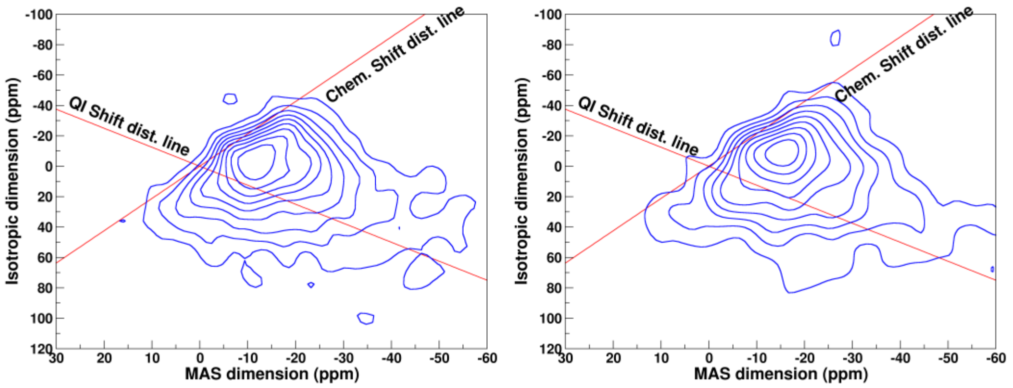

Figure S6: ^{23}Na MQMAS spectrum of (a) the ISG-Cm Annealed glass (7 10^{16} α/g) and (b) the ISG-Cm Damaged glass (5.8 10^{18} α/g).

Glass	δ_{iso} (ppm)	Cq (MHz)	η
ISG-Cm Annealed 7 10^{16} α/g	-7 (6)	2.7 (0.9)	0.6 (0.3)
ISG-Cm Damaged 4.4 10^{18} α/g	-4 (6)	2.9 (1.0)	0.6 (0.3)
ISG	-11 (8)	2.5 (0.8)	0.6 (0.3)
ISG Damaged Xe	-7 (6)	2.9 (1.0)	0.6 (0.3)
ISG Damaged Au	-4 (10)	3.0 (1.0)	0.6 (0.3)

Table S3: Mean values of ^{23}Na NMR parameter distribution used to analyze the ^{23}Na MAS NMR spectra shown in Fig. S5. Standard deviation values (i.e. width of the NMR parameters distribution) are given in parentheses. δ_{iso}: isotropic chemical shift; Cq: Quadrupolar coupling constant; η: quadrupolar asymmetry parameter

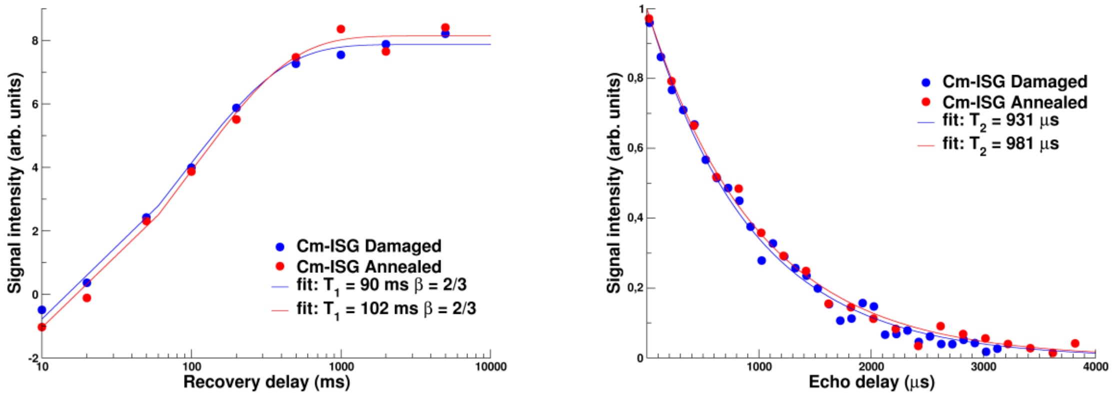

Figure S7: Longitudinal (left panel, using inversion-recovery pulse sequence) and transverse (right panel, using spin echo) ^{23}Na relaxation times under static (i.e. non spinning) conditions with pulses selective on the central transition (-½ , ½). Inversion-Recovery and Spin-Echo curves were fitted with a stretched exponential $M(t) = M_\infty \times \left(1 - e^{-\left(\frac{t}{T_1}\right)^\beta}\right)$ and a single decaying exponential function $(t) = M(0) \times e^{-\frac{t}{T_2}}$, respectively.

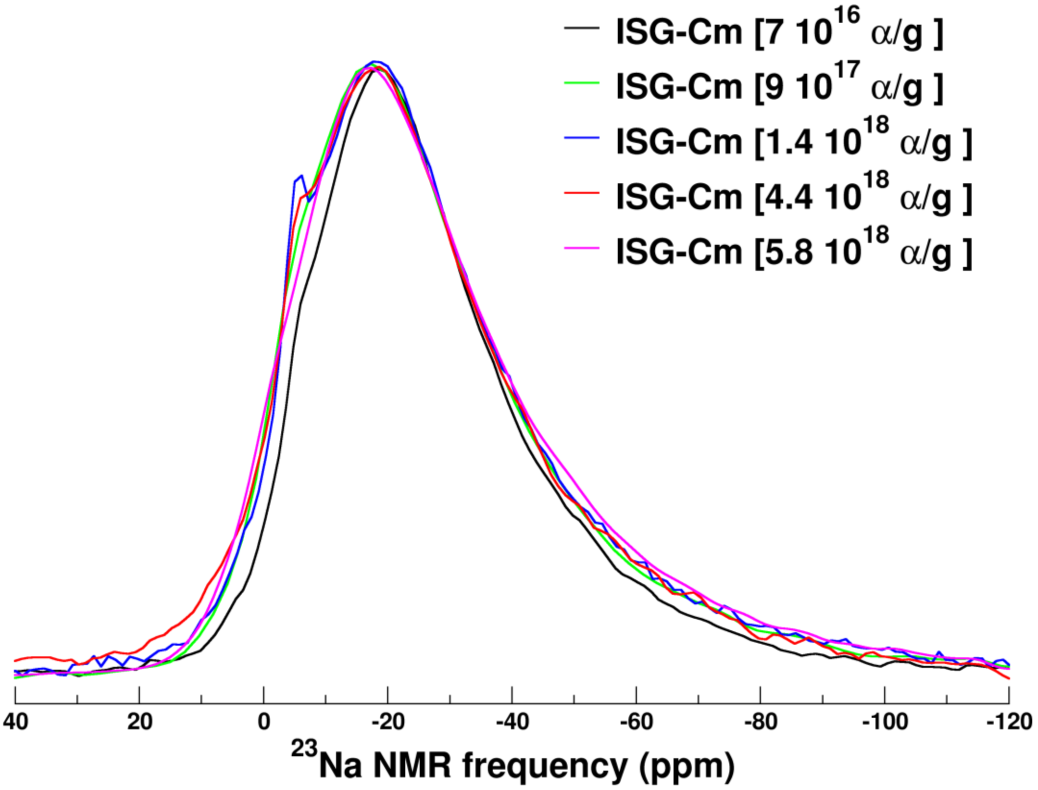

Figure S8: ^{23}Na MAS NMR spectra of the ISG-Cm glasses with increasing alpha decay dose.

4. ^{29}Si MAS NMR

^{29}Si MAS NMR spectra were acquired using the CPMG pulse sequence with 180° pulse width of 8 μs and echo delays of 4ms. Typically 10 echoes were accumulated in 4096 scans with a recycle delay of 2s. No change in lineshape was observed for a longer recycle delay of 20s (256 scans). Echoes were coadded, followed by Gaussian apodization of 100 Hz before Fast Fourier Transform.

^{29}Si spectra were acquired using a rotor-synchronized Carr–Purcell–Meiboom–Gill (CPMG) echo techniques [6]. The spectra consist of a single broad peak which is due to an overlap of several $Si(OSi)_n(O^-)_{4-n}$ units, in addition to effects of Si-O-B and Si-O-Al connectivities. Considering the NMR shifts and previous ^{17}O NMR studies that corroborated low content of non-bridging oxygen for this type of glasses [7, 8], two types of SiO_4 tetrahedra with respectively three and four bridging oxygen atoms, are mainly observed (Fig. S9). No spectral are modifications observed with self-irradiation time.

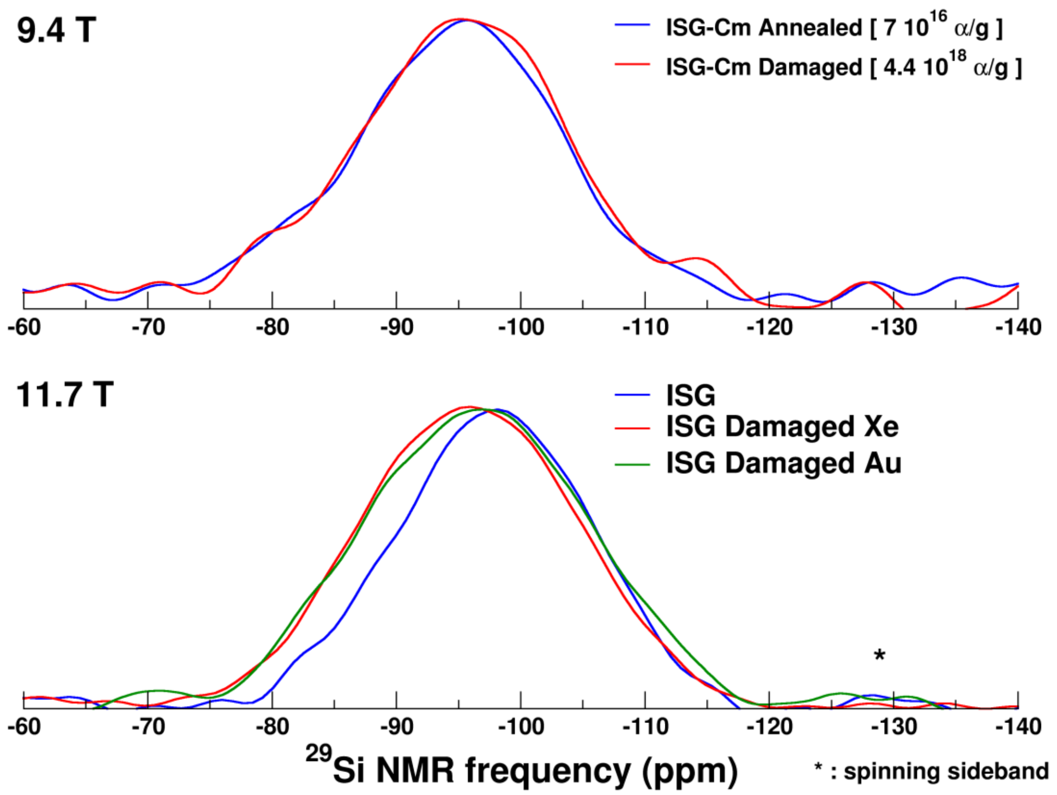

Figure S9: ^{29}Si MAS NMR spectra of (Upper panel) ISG-Cm glasses and (Lower panel) ISG and ISG damaged glasses irradiated by Xenon [3] and Gold [4] irradiations. Spectra are normalized to the same maximum height.

5. ^{27}Al MAS NMR

^{27}Al MAS NMR spectra were acquired with single pulse excitation of short length (1μs, tip angle ~ 10-20°) to ensure quantitatively and with a recycle delay ensuring a full relaxation of the nuclear spins (0.5s). Typically between 2048 and 8192 scans were accumulated to obtained good signal to noise ratio. Multiple Quantum MAS (MQMAS) spectra were collected using the three-pulse Z-filter pulse sequence [1] with pulse widths of 2μs and 1μs for the first two Triple Quantum pulses (RF field of ~160 kHz), and a third 90° pulse of 2μs (RF field 42kHz). Typically 1200 transients were accumulated per t1-value with 40 rotor-synchronized t1 increments (20μs) at a recycle delay of 0.25s.

^{27}Al MAS (Fig. 2) and MQMAS (Fig. S10) were performed on the two references glasses (ISG-Cm glasses at the beginning if this study) and with time evolution. As confirmed by the MQMAS experiments, the spectra of ISG-Cm Damaged (4.4 10^{18} α/g) is defined by a single broad peak, here centered at about 60 ppm, and no contribution from higher coordinated species (AlO_5 and AlO_6) could be observed. The spectra were therefore analyzed using NMR parameter distributions as described in Ref. [1] yielding NMR parameters gathered in Table S4. As shown in Fig. S11, no significant change in the ^{27}Al NMR transverse and longitudinal relaxation times were observed under static (i.e. non spinning sample) conditions. Interestingly, even under

static conditions, a significant difference could be observed as well between the two initial glasses (Fig. S12).

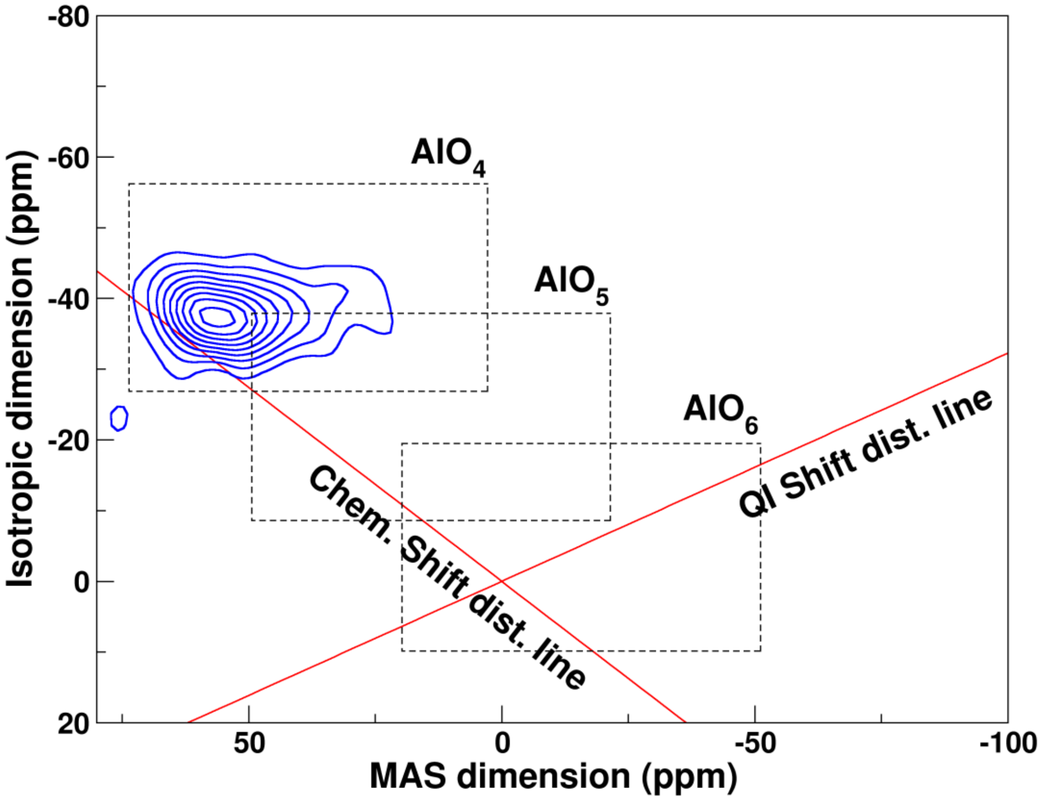

Figure S10: ^{27}Al MQMAS spectrum of the ISG-Cm Damaged glass (4.4 10^{18} α/g). Dashed-line boxes show typical peak position of AlO_x site. Red lines show broadening direction as induced by isotropic chemical shift distribution (Chem. Shift. Dist. Line) and quadrupolar interaction distribution (Second Order Quadrupolar Induced – here denoted QI – Shift dist. Line).

Glass	δ_{iso} (ppm)	C_Q (MHz)	η
ISG-Cm Annealed 7 10^{16} α/g	62 (4)	4.2 (1.8)	0.6 (0.3)
ISG-Cm Damaged 4.4 10^{18} α/g	63 (5)	5.8 (2.5)	0.6 (0.3)
ISG	61 (5)	4.7 (1.6)	0.6 (0.3)
ISG Damaged Xe	64 (6)	6.4 (2.1)	0.6 (0.3)
ISG Damaged Au	65 (7)	6.5 (2.1)	0.6 (0.3)

Table S4: Mean values of ^{27}Al NMR parameter distribution used to analyze the ^{27}Al MAS NMR spectra (AlO_4 site). Standard deviation values are given in parentheses. δ_{iso} : isotropic chemical shift; C_Q : Quadrupolar coupling constant; η : quadrupolar asymmetry parameter.

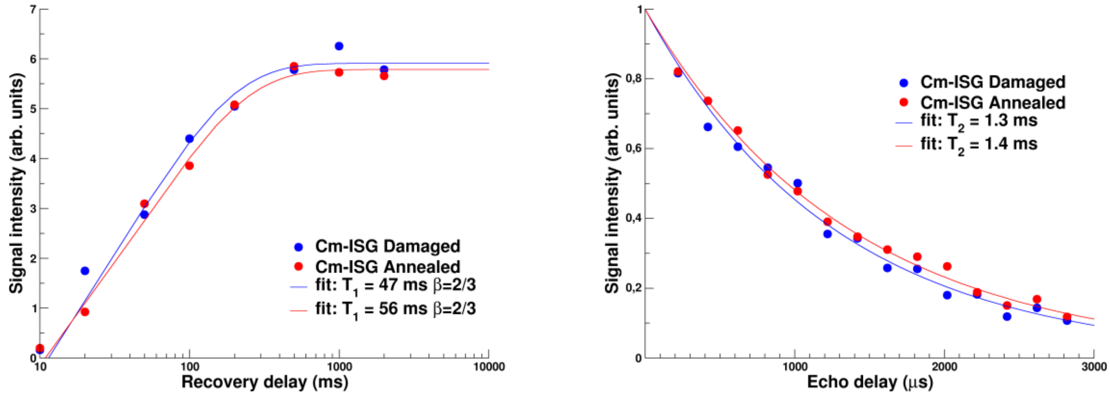

Figure S11: Longitudinal (left panel, using inversion-recovery pulse sequence) and transverse (right panel, using spin echo) ^{27}Al relaxation times under static (i.e. non spinning) conditions with pulses selective on the central transition (-½ , ½). Inversion-Recovery and Spin-Echo curves were fitted with a stretched exponential $M(t) = M_\infty \times \left(1 - e^{-\left(\frac{t}{T_1}\right)^\beta}\right)$ and a single decaying exponential function $(t) = M(0) \times e^{-\frac{t}{T_2}}$, respectively.

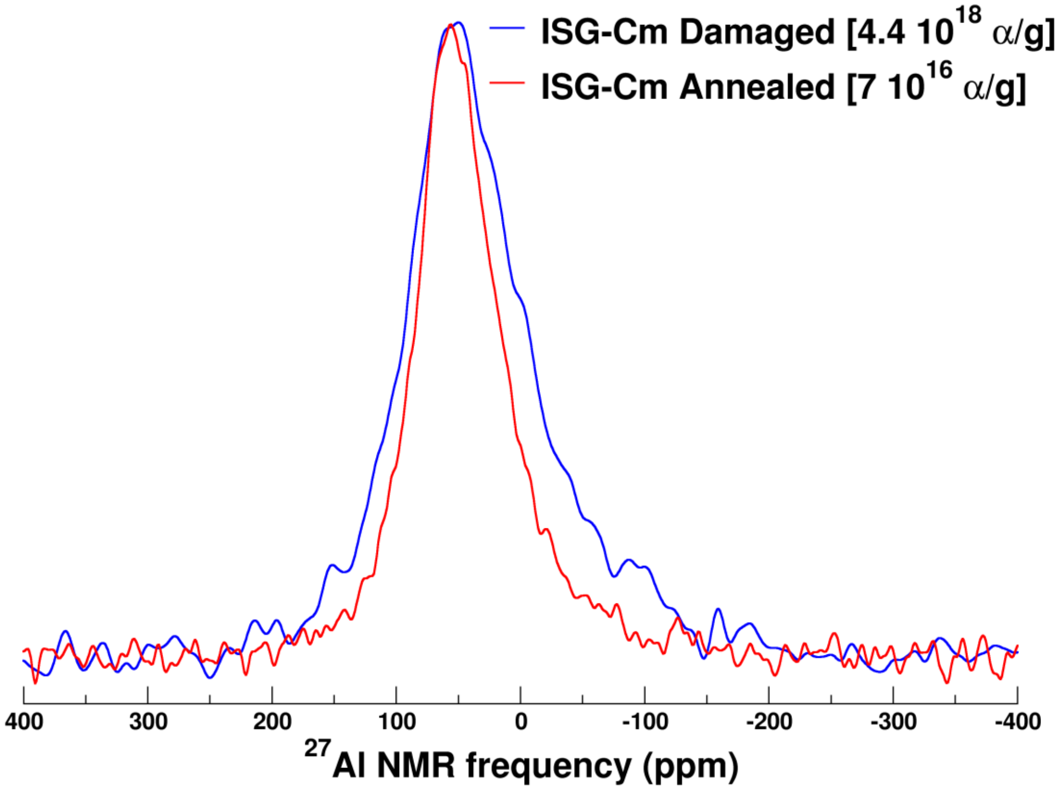

Figure S12: ^{27}Al static (i.e. non spinning sample) spectra of the ISG-Cm glasses.

References

[1] F. Angeli, T. Charpentier, D. de Ligny, and C. Cailleteauz, "Boron Speciation in Soda-Lime Borosilicate Glasses Containing Zirconium," *Journal of American Ceramic Society,* vol. 93, pp. 2693-2704, Sep 2010.

[2] S. Peuget, E. A. Maugeri, T. Charpentier, C. Mendoza, M. Moskura, T. Fares*, et al.*, "Comparison of radiation and quenching rate effects on the structure of a sodium borosilicate glass," *Journal of Non-Crystalline Solids,* vol. 378, pp. 201-212, 2013.

[3] C. Mendoza, S. Peuget, T. Charpentier, M. Moskura, R. Caraballo, O. Bouty*, et al.*, "Oxide glass structure evolution under swift heavy ion irradiation," *Nuclear Instruments and Methods in Physics Research Section B: Beam Interactions with Materials and Atoms,* vol. 325, pp. 54-65, 4/15/ 2014.

[4] S. Peuget, J. M. Delaye, and C. Jégou, "Specific outcomes of the research on the radiation stability of the French nuclear glass towards alpha decay accumulation," *Journal of Nuclear Materials,* vol. 444, pp. 76-91, 1// 2014.

[5] H. B. Du, V. V. Terskikh, C. I. Ratcliffe, and J. A. Ripmeester, "Distinguishing surface versus buried cation sites in aluminosilicate mesoporous materials," *Journal of the American Chemical Society,* vol. 124, pp. 4216-4217, Apr 24 2002.

[6] F. H. Larsen and I. Farnan, "Si-29 and O-17 (Q)CPMG-MAS solid-state NMR experiments as an optimum approach for half-integer nuclei having long T-1 relaxation times," *Chemical Physics Letters,* vol. 357, pp. 403-408, May 17 2002.

[7] F. Angeli, T. Charpentier, E. Molieres, A. Soleilhavoup, P. Jollivet, and S. Gin, "Influence of lanthanum on borosilicate glass structure: A multinuclear MAS and MQMAS NMR investigation," *Journal of Non-Crystalline Solids,* vol. 376, pp. 189-198, Sep 15 2013.

[8] F. Angeli, T. Charpentier, M. Gaillard, and P. Jollivet, "Influence of zirconium on the structure of pristine and leached soda-lime borosilicate glasses: Towards a quantitative approach by O-17 MQMAS NMR," *Journal of Non-Crystalline Solids,* vol. 354, pp. 3713-3722, Aug 1 2008.
